# Projected marine climate change: effects on copepod oxidative status and reproduction

**DOI:** 10.1002/ece3.839

**Published:** 2013-10-21

**Authors:** Anu Vehmaa, Hedvig Hogfors, Elena Gorokhova, Andreas Brutemark, Towe Holmborn, Jonna Engström-Öst

**Affiliations:** 1 ARONIA Coastal Zone Research Team, Novia University of Applied Sciences & Åbo Akademi UniversityEkenäs, Finland; 2 Tvärminne Zoological Station, University of HelsinkiHanko, Finland; 3 Department of Ecology, Environment and Plant Sciences, Stockholm UniversityStockholm, Sweden; 4 Department of Applied Environmental Science, Stockholm UniversityStockholm, Sweden; 5 Calluna ABStockholm, Sweden

**Keywords:** antioxidant capacity, copepod egg production, hatching success, nauplii development, ocean acidification, oxidative stress, toxic algae, warming

## Abstract

Zooplankton are an important link between primary producers and fish. Therefore, it is crucial to address their responses when predicting effects of climate change on pelagic ecosystems. For realistic community-level predictions, several biotic and abiotic climate-related variables should be examined in combination. We studied the combined effects of ocean acidification and global warming predicted for year 2100 with toxic cyanobacteria on the calanoid copepod, *Acartia bifilosa*. Acidification together with higher temperature reduced copepod antioxidant capacity. Higher temperature also decreased egg viability, nauplii development, and oxidative status. Exposure to cyanobacteria and its toxin had a negative effect on egg production but, a positive effect on oxidative status and egg viability, giving no net effects on viable egg production. Additionally, nauplii development was enhanced by the presence of cyanobacteria, which partially alleviated the otherwise negative effects of increased temperature and decreased pH on the copepod recruitment. The interactive effects of temperature, acidification, and cyanobacteria on copepods highlight the importance of testing combined effects of climate-related factors when predicting biological responses.

## Introduction

Zooplankton are the major link between pelagic primary producers and fish (Mauchline 1998). Therefore, when predicting effects of climate change on pelagic ecosystems, it is crucial to address zooplankton responses. Most studies have investigated the isolated effects of temperature increase (Richardson 2008) or ocean acidification (Fabry et al. 2008), whereas some have considered interactions between a plethora of biotic and abiotic climate-related variables (Byrne et al. 2009; Gao and Zheng 2010; Vehmaa et al. 2012; Kroeker et al. 2013; Reymond et al. 2013). Bloom-forming harmful algal species, such as cyanobacteria, are promoted by eutrophication. Further, rising temperature enhances stratification, which is favorable to these species (Hallegraeff 2010) and can consequently stimulate harmful blooms of cyanobacteria. As some cyanobacteria can have negative effects on grazers due to their toxin production and poor nutritional properties (Porter and Orcutt 1980), increased toxic cyanobacteria could impact zooplankton grazers and functioning of the whole pelagic ecosystem, particularly when combined with acidification and warming. It is therefore essential to understand combined effects of cyanobacteria and climatic factors on zooplankton.

Elevated CO_2_ in the oceans affects calcifying marine animals, such as corals, molluscs, and echinoderms, by interrupting fertilization, calcification, growth, and survival (Kroeker et al. 2010, 2013; Pandolfi et al. 2011; Branch et al. 2013). Effects of acidification on lightly calcified species, such as planktonic crustaceans, are less known. Meta-analyses suggest that crustaceans in general are less sensitive to acidification than heavily calcified organisms (Kroeker et al. 2010, 2013). Other studies show that the ability of crustaceans to adjust acid-base imbalances during high CO_2_ exposure could be metabolically expensive and affect growth and survival (reviewed by Whiteley 2011; Fitzer et al. 2012).

The evidence is also accumulating that many metabolic responses and physiological disorders caused by suboptimal pH levels are related to oxidative stress (Todgham and Hofmann 2009; Tomanek et al. 2011; Kaniewska et al. 2012), which is the imbalance between pro-oxidant and antioxidant homeostatic cellular conditions associated with increased production of nitrogen or oxygen reactive species and resulting in accumulation of lipid peroxidation products and increased oxidation of proteins and DNA. To counteract these detrimental effects of increased reactive species production, cells possess a powerful and complex antioxidant defense system composed of various antioxidants. When the production of reactive species increase, cells stimulates antioxidants production and/or their activity, which hampers pro-oxidative processes and prevents cell damage (review by Pamplona and Costantini 2011).

Increases in antioxidant enzymes are commonly reported in controlled laboratory studies of oxidative stress in aquatic animals imposed by both chemical and physical agents (Choi et al. 2000). However, activation of an antioxidant response may be energetically and nutrient demanding (Pamplona and Costantini 2011), and high stress level (or additional stressors) has been reported to exacerbate oxidative stress and exhaust antioxidative capacity resulting in decreased antioxidant levels (Gorokhova et al. 2010, 2013a). In line with this, expression of genes involved in maintaining protein integrity and defending against oxidative stress is lower in sea urchin larvae under moderate and high CO_2_-driven acidification (Todgham and Hofmann 2009). Additionally, oxidative stress is emerging as a common denominator in complex response mechanisms to climate change (e.g., warming, UV, brownification; reviewed by Lesser 2006; Tomanek 2011) and algal toxins (reviewed by Amado and Monserrat 2010) in a variety of aquatic organisms. In ecology and ecotoxicology, most specific tests for oxidative stress evaluation are based on the general principle of assaying at least two of the four components of oxidative stress (free radical production, antioxidant defenses, oxidative damage, and repair mechanisms), with measurements of antioxidant defenses and oxidative damage being most popular (Monaghan et al. 2009).

Based on these and other studies, we hypothesized that in copepods (1), short-term exposure to the pH levels predicted by the 2100 climate scenario (between 0.2 and 0.4 pH units lower; Caldeira and Wickett 2003; IPCC 2007) would negatively impact oxidative status, with little or no immediate effects on the reproductive output (Kurihara et al. 2004; Mayor et al. 2007); (2) higher temperature (i.e., +3–5°C projected for the Baltic Sea basin during this century; HELCOM 2007) would negatively affect oxidative status (Metcalfe and Alonso-Alvarez 2010) and result in decreases in egg production, egg viability, and nauplii development (Koski and Kuosa 1999; Chinnery and Williams 2004); (3) the pH effect would increase species sensitivity to temperature (Pörtner and Farrell 2008); and (4) pH and temperature effect would also be enhanced when combined with another stress factor, the presence of the toxic cyanobacterium *Nodularia spumigena,* which is one of the dominating bloom-forming species in the study area, the Baltic Sea (Wasmund et al. 2011). The latter hypothesis is based on numerous observations that cyanobacterial secondary metabolites are capable of inducing oxidative stress in variety of organisms (Amado and Monserrat 2010).

To test these hypotheses, we conducted an experiment which consisted of eight treatments representing combinations of ambient (17°C) or higher (20°C) temperature, ambient (∼8.0) or lower (∼7.6) pH, and different diets consisting of either green algae or green algae mixed with the toxic cyanobacteria *N. spumigena* (9:1 by biomass). Dissolved inorganic carbon (DIC) and total nodularin concentration (a hepatotoxin produced by *N. spumigena*) were used as explanatory variables in statistical analysis for acidity/pH and presence of cyanobacteria, respectively. Ambient values refer to ambient surface water values in the area at the time of the study. As a model species, we used a common Baltic copepod *Acartia bifilosa*. We analyzed its reproductive parameters (egg production rate, egg viability, viable egg production rate, and development index) and oxidative status (antioxidant capacity, oxidative damage, and oxidative balance expressed as a ratio between the antioxidant capacity and the oxidative damage).

## Material and Methods

### Test organisms

The cyanobacterium, *Nodularia spumigena* (AV1), was grown in Z8 medium (Kótai 1972) without nitrogen. The chlorophyte strain, *Brachiomonas submarina* (TV15, Tvärminne Zoological Station, University of Helsinki), was grown in f/2 (without silica) medium according to Guillard (1975). Both cultures were nonaxenic batch monocultures and kept at 18°C under 16:8 h light:dark cycle and 13.7 μmol photons m^−2^ s^−1^.

**Figure 1:**
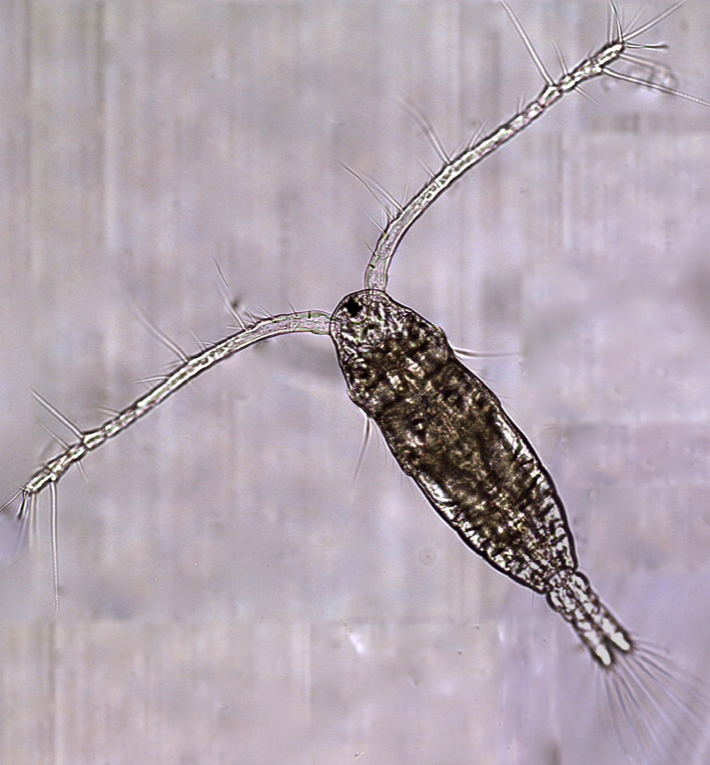
Adult female of the calanoid copepod *Acartia bifilosa*.

Adult (copepodite stage VI) calanoid copepods (*Acartia bifilosa*) were collected in 8th August 2010 with a 150 μm net by vertical hauls at Storfjärden (59°51′20′′N, 23°15′42′′E; near Tvärminne Zoological Station, Finland; Fig. 1).

### Experimental set-up

**Figure 2:**
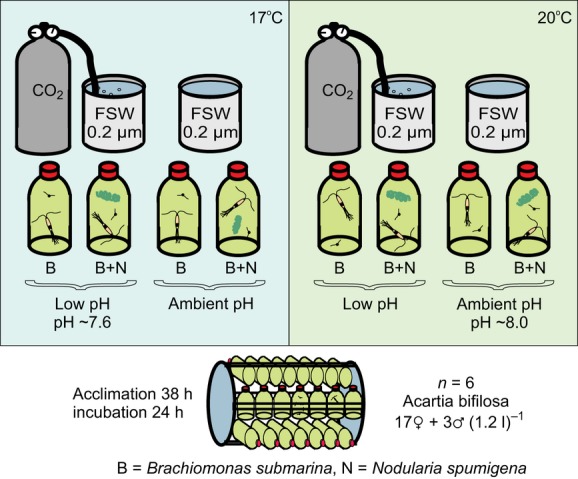
Experimental set-up. *Acartia bifilosa* copepods were incubated in 1.2 l bottles on plankton wheels. The bottles containing the animals and filtered sea water (FSW) were exposed to 17°C or 20°C temperature, ambient, or CO_2_-lowered pH conditions and diet containing chlorophyte *Brachiomonas submarina* (B) or 9:1 mixture of *B. submarina* and toxic cyanobacterium *Nodularia spumigena* (B+N). After 38 h acclimation and 24 h incubation periods, copepod oxidative status and reproductive success were measured.

Each of the eight treatments was replicated six times (Fig. 2). Low pH treatments (∼7.6) were adjusted by aerating filtered seawater (Sartobran P 300 0.2 μm; collected at 10 m depth from Storfjärden; salinity 5.7; ambient pH ∼8.0) with industrial CO_2_ gas using a TUNZE pH/CO_2_-controller-set (7074/2). The 1.2 l bottles, each containing 17 female and 3 male copepods, were incubated on plankton wheels rotating at 1 rpm at an irradiance of 18.2 ± 2.5 μmol photons m^−2^ s^−1^ on top of the wheel for 16 h a day. Copepods were fed at surplus, using either monoculture of *B. submarina* (1061 ± 87 μg C L^−1^; average ± SD) or a mixture of *B. submarina* (971 ±208 μg C L^−1^) and *N. spumigena* (102 ± 17.7 μg C L^−1^). Treatments with *N. spumigena* had the same nodularin concentration (1-ANOVA: *F*_3,20_ = 1.26, *P* = 0.315), on average (± SD) 1.49 ± 0.35 μg L^−1^. After a 36 h acclimation period, the media was changed, and the copepods were incubated for another 24 h at the same conditions. At the end of the experiment, the copepods from each bottle were filtered through a 120 μm mesh and counted under a dissecting microscope. Live copepods from two replicate bottles were pooled onto a plankton net (diameter ∼1.5 cm, mesh size 45 μm), which was stored in Eppendorf tubes at −80°C; these samples were used for antioxidant capacity and oxidative damage (lipid peroxidation) measurements. Ambient temperature and pH values in the current study refer to ambient temperature and pH after filtration in the water that was sampled from 10 m depth at the time of the study.

### Egg production, egg viability, viable egg production, and development index

Eggs and nauplii from each bottle were collected with a 48 μm mesh into a petri dish and stored overnight in the dark at 3°C. From this pool, ∼50 eggs bottle^−1^ were taken for egg viability (% viable eggs) analysis, and the remaining eggs and nauplii were preserved with acid Lugol's solution to estimate egg production and the development index. Egg viability was estimated by staining with TO-PRO-1 iodide (Molecular Probes) which only penetrates eggs with damaged plasma membrane (Gorokhova 2010). The development index was calculated according to Knuckey et al. (2005):


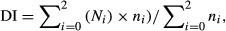


where *N*_*i*_ is the assigned stage value (*N*_0_ [egg] = 0; *N*_1_ = 1; *N*_2_ = 2) and *n*_*i*_ is the number of copepods at that stage. No nauplii older than *N*_2_ were found; thus, they were nonfeeding (Baud et al. 2002). Egg production rate (eggs female^−1^ day^−1^) was calculated using all eggs and nauplii. To estimate egg viability, the number of viable eggs and nauplii in the egg viability analysis was first calculated:


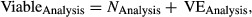


where Viable_Analysis_ = viable eggs and nauplii, *N*_Analysis_= nauplii, and VE _Analysis_ = viable eggs in the egg viability analysis. Secondly, the number of nauplii and projected number of viable eggs in the lugol preservation (for DI-analysis) was estimated:


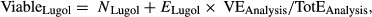


where Viable_Lugol_ = viable eggs and nauplii, *N*_Lugol_ = nauplii and *E*_Lugol_ = eggs in the lugol preservation and TotE_Analysis_ = total number of eggs in the egg viability analysis. Finally, egg viability was calculated:


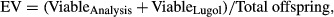


where viable eggs and nauplii in both the egg viability analysis and the lugol-preserved sample were divided with total offspring from each incubation.

### pH and DIC

In each bottle, pH was measured (TUNZE pH-controller 7070/2) shortly before the incubation and immediately after the bottles were opened after the incubation. Samples for total dissolved inorganic carbon (DIC; carbon dioxide, carbonic acid, bicarbonate ions and carbonate ions) were taken from each treatment water batch before incubation and from each bottle after incubation. Samples were kept in air-free vials in darkness and on ice until measured 48 h later using acidification/gas stripping/infrared detection method (Salonen 1981; Dickson 2010). In ambient pH treatments (∼8.0), DIC was 1860 ± 25 μmol L^−1^ (average ± SD) and in low pH treatments (∼7.6) 1950 ± 22 μmol L^−1^ (average ± SD).

### Cyanobacteria

Lugol-preserved samples containing *N. spumigena* were counted at 400× magnification under an inverted Leica ILRB microscope (Leica, Wetzlar, Germany) (Utermöhl 1958). At least 200 cells per sample were counted. Particulate organic carbon (POC) was measured from each bottle and from the phytoplankton cultures, and determined with a mass spectrometer (Elemental Combustion System CHNS-O 4010; Uppsala University, Sweden). *N. spumigena* cell counts were converted to carbon using the POC measurements from the culture.

Nodularin concentrations (intracellular and extracellular) were analyzed with ELISA, using a microcystin plate kit (EnviroLogix, Portland, ME, USA) and microcystin standards (0.16–2.5 ng mL^−1^) according to the kit instructions (Metcalf and Codd 2003).

### Copepod oxidative status

The copepods recovered from the filters were transferred to microcentrifuge tubes containing 0.7 ml of PBS, homogenized for 4 min using FastPrep with cooling function and 100 μm glass beads, and centrifuged at 10 000 ***g*** for 5 min at 4°C. Intracellular soluble antioxidant capacity was measured from the homogenized samples using the oxygen radical absorption capacity (ORAC) assay (Prior et al. 2003). This assay has been used to measure antioxidative status in amphipods (Gorokhova et al. 2013a) and copepods (Gorokhova et al. 2013b) and correlate well with standard antioxidant biomarkers, such as superoxide dismutase (SOD) and catalase (CAT; Gorokhova et al. 2013a). Oxidative damage was measured by the lipid peroxidation assay using QuantiChrom™ TBARS Assay Kit (thiobarbituric acid reactive substances; TBARS; DTBA-100; BioAssay Systems, USA) as specified by the manufacturer. Water soluble protein concentration (μg mL^−1^) was measured by the bicinchoninic acid assay (BCA, Pierce Ltd.) with bovine serum albumin (BSA, 1–5 μg well^−1^) as standard following directions of the manufacturer for microtiter assays.

### Data analysis/statistics

General linear model (GLM) and Spearman rank-order correlations, with STATISTICA 10 (StatSoft, Inc., 2010), were used for the statistical analysis. To test our hypotheses, we used backward stepwise GLM where the oxidative status variables (ORAC, TBARS, ORAC:TBARS) and reproductive output (egg production rate, egg viability, viable egg production rate and development index) were used as dependent variables, and temperature (2-level factor), dissolved inorganic carbon (indicative of pH), the cyanobacterial toxin nodularin (intracellular + extracellular) were used as independent variables. For GLMs, Box–Cox and Freeman–Tukey transformations were used for the egg viability and the development index, respectively. Visual inspection of distribution plots and Kolmogorov–Smirnov and Lilliefors tests were used to confirm that there was no multicollinearity, heteroscedasticity in the residuals, or deviations from normal distribution of the residuals. Spearman rank correlations were used to test the correlations between repro-ductive output variables and oxidative status variables. 1-ANOVA was used to check whether there are differences in toxicity between the treatments containing *N. spumigena*.

## Results

**Figure 3:**
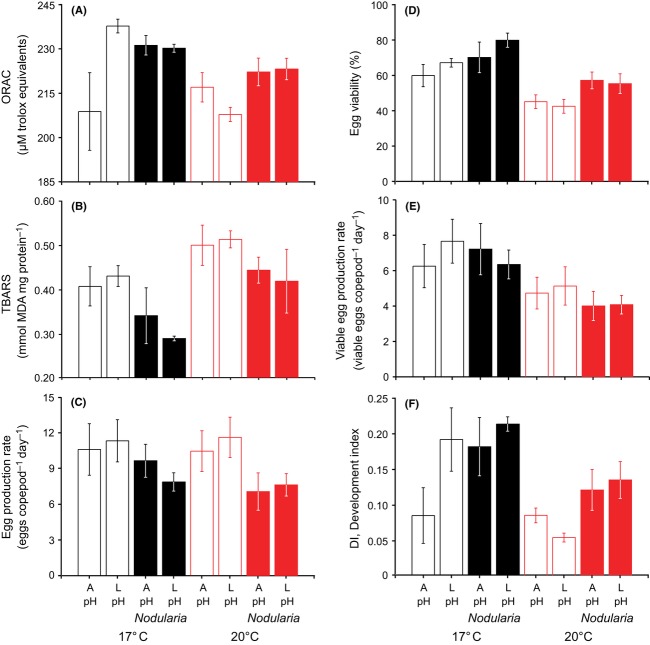
Copepod (A) antioxidant capacity [ORAC (μmol L^−1^ trolox equivalents)], (B) oxidative damage [lipid peroxidation levels; TBARS (mmol MDA mg protein^−1^)], (C) egg production rate, (D) egg viability, (E) viable egg production rate, and (F) nauplii development index in different treatments. N = 3 for panels (A) and (B), and N = 6 for panels (C), (D), (E) and (F). Average ± SE. “A pH” denotes ambient pH (∼8.0) and “L pH” low pH (∼7.6). Treatments with open bars had *Brachiomonas submarina* as sole food; filled bars mean that the feeding media included *Nodularia spumigena* (10% of the total food concentration). Black color indicates 17°C (ambient temperature) and red color 20°C (high temperature).

Acidification did not have any significant direct effects on either oxidative status variables or reproductive output variables (Fig. 3). However, a significant interaction effect of acidification and temperature on antioxidant capacity was found, which means that the pH effect is temperature specific (Table 1). At 17°C, acidification increased antioxidant capacity, whereas at 20°C, acidification decreased the antioxidant capacity (Fig. 3A). Moreover, higher temperature had a significant positive effect on antioxidant capacity and oxidative damage, but a negative effect on oxidative balance measured as a ratio of antioxidant capacity and oxidative damage. Also, higher temperature had a negative effect on egg viability, viable egg production rate, and nauplii development, but no significant effect on egg production rate (Figs 3C–F).

**Table 1:** General linear models. The dependent variables of oxidative balance were as follows: antioxidant capacity (ORAC), oxidative damage (TBARS), and oxidative balance (ORAC:TBARS ratio); and of reproductive parameters: egg production rate, egg viability, viable egg production rate, and development index. As explanatory variables, temperature, DIC (Dissolved inorganic carbon), and the cyanobacterial toxin nodularin were used. DIC and the toxin nodularin were measurements for acidification and presence of cyanobacteria, respectively. Only significant models are shown

Model/Variable	Beta ± SE	*P* value	*R*^2^	Adjust *R*^2^	SE estimate
Antioxidant capacity					
Model		<0.001	0.35	0.31	11
Temperature 20°C	14 ± 5.2	0.012			
Temp 20°C *DIC	−14 ± 5.2	0.01			
Toxin nodularin	0.28 ± 0.12	0.027			
Oxidative damage					
Model		<0.001	0.41	0.38	0.078
Temperature 20°C	0.54 ± 0.12	<0.001			
Toxin nodularin	−0.37 ± 0.12	0.002			
Oxidative balance					
Model		<0.001	0.45	0.43	130
Temperature 20°C	−0.54 ± 0.11	<0.001			
Toxin nodularin	0.43 ± 0.11	<0.001			
Egg production rate					
Model		0.006	0.15	0.13	3.6
Toxin nodularin	−0.39 ± 0.14	0.006			
Egg viability^1^					
Model		<0.001	0.47	0.45	0.1
Temperature 20°C	−0.61 ± 0.11	<0.001			
Toxin nodularin	0.36 ± 0.11	0.002			
Viable egg production rate					
Model		0.002	0.2	0.18	2.4
Temperature 20°C	−0.45 ± 0.13	0.002			
Development index^2^					
Model		0.004	0.22	0.18	0.16
Temperature 20°C	−0.35 ± 0.13	0.011			
Toxin nodularin	0.33 ± 0.13	0.016			

1 Box–Cox transformed.

2 Freeman–Tukey transformed.

Nodularin had a positive effect on antioxidant capacity and a negative effect on oxidative damage, which resulted in a positive effect on oxidative balance (Table 1). Moreover, presence of cyanobacteria had positive effect on egg viability and development index (Table 1; Figs 3D, F). However, the egg production rate was negatively affected (Table 1; Fig. 3C). No cyanobacteria effect was detected for the viable egg production rate (Table 1; Fig. 3E).

**Figure 4:**
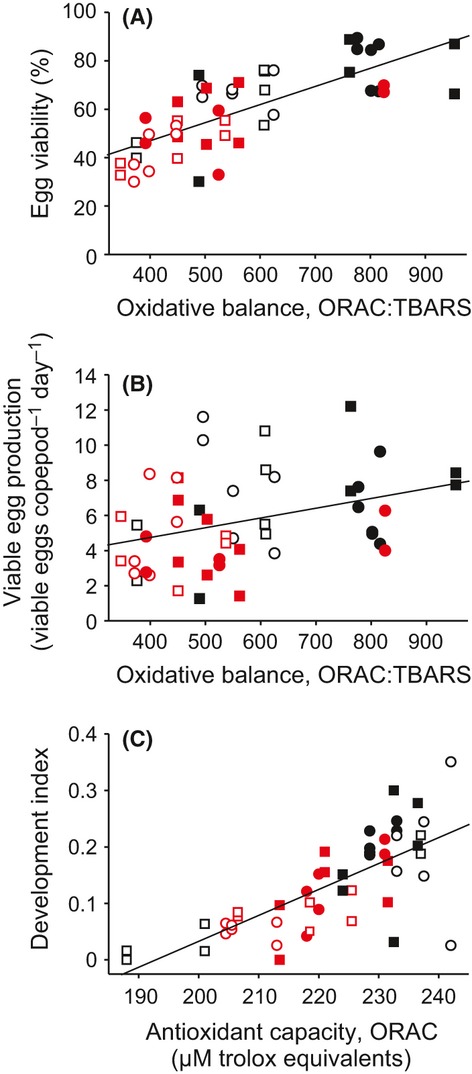
Scatterplot and a linear fit between copepod (A) egg viability and maternal oxidative balance measured as a ratio of antioxidant capacity [ORAC (μmol L^−1^ trolox equivalents)] and oxidative damage [lipid peroxidation levels; TBARS (mmol MDA mg protein^−1^)], (B) viable egg production and oxidative balance, and (C) development index and antioxidant capacity [ORAC (μmol L^−1^ trolox equivalents)]. Treatments with ambient pH (∼8.0) are plotted as square symbols and treatments with low pH (∼7.6) as circles. Treatments with open symbols had *Brachiomonas submarina* as sole food; filled symbols mean that the feeding media included *Nodularia spumigena* (10% of the total food concentration). Black color indicates 17°C (ambient temperature) and red color 20°C (high temperature).

No significant correlations were revealed for egg production rate when tested against oxidative status variables (Spearman rank correlation: *P* > 0.05). Egg viability was significantly correlated (*P* < 0.05) to all oxidative status variables, most strongly with oxidative balance (Fig. 4A) and oxidative damage, with positive (*R* = 0.75) and negative correlations (*R* = −0.73), respectively, and slightly less with antioxidant capacity (*R* = 0.54). Viable egg production rate had a weak but significant (*P* < 0.05) correlations with all three oxidative status variables (antioxidant capacity *R* = 0.34, oxidative damage *R* = −0.33; oxidative balance *R* = 0.35, Fig. 4B). Development index had the highest correlation with antioxidant capacity (*R* = 0.72, *P* < 0.05, Fig. 4C). Also oxidative damage (*R* = −0.44) and oxidative balance (*R* = 0.52) correlated significantly (*P* < 0.05) with the development index.

## Discussion

We have investigated the effects of three environmental factors associated with climate change: acidification, temperature increase, and toxic cyanobacteria on copepod oxidative status and reproduction. In agreement with other studies (Kurihara et al. 2004; Mayor et al. 2007), we did not find significant acidification effects on reproductive parameters in a pH scenario projected for the year 2100. However, we found a significant acidification-dependent effect in interaction with temperature on the antioxidant capacity, suggesting that in warmer oceans, antioxidant defenses might not be adequate, which increases risk of oxidative stress in copepods (and possibly other organisms). This is in line with the lower expression of a number of genes involved in defending against oxidative stress found in sea urchins under CO_2_ driven acidification (Todgham and Hofmann 2009). Temperature increase by 3°C had strong negative effects on copepods: the oxidative balance shifted toward pro-oxidative processes, eggs viability decreased, and the nauplii developed more slowly.

The significant interaction effect of acidification and temperature on the antioxidant capacity that increased at ambient temperature and decreased at high temperature (Fig. 3A) indicates that at intermediate stress levels (i.e., at low pH and ambient temperature), an upregulation of the antioxidant system enhances protection against oxidative damage. However, at higher stress (i.e., when the temperature is high, both in conjunction with ambient pH but especially in conjunction with low pH), the pro-oxidants may exceed the capacity of the antioxidant system and lead to oxidative damage (Fig. 3A, B). The observed antioxidant capacity response suggests that when combined with elevated temperature, copepods increase antioxidant defenses already at pH lowered by 0.4.

Although antioxidant capacity increased with temperature, oxidative balance was negatively affected due to the cumulative oxidative damage at the high temperature (Table 1). These results indicate that copepod oxidative balance shifts towards a pro-oxidative state at temperatures above the species optimum (13–18°C; Koski and Kuosa 1999) and leads to lowered egg viability, decreased viable egg production, and slowed nauplii development.

Contrary to our hypothesis, the presence of cyanobacteria promoted antioxidative defenses and decreased lipid peroxidation levels (oxidative damage), thus contributing toward maintenance of the redox-state and oxidative balance of the copepods (Table 1; Fig. 3A, B), which are similar to the effects found in estuarine crabs exposed to another hepatotoxin, microcystin (Pinho et al. 2005). However, in crabs, the increased activity of the antioxidant enzymes was not associated with a decreased oxidative damage as in the present study. In contrast, other studies indicate that hepatotoxins may decrease the antioxidant activity (Lankoff et al. 2002) or elevate the formation of reactive oxygen species leading to oxidative damage (Amado and Monserrat 2010). In the study of Jiang et al. (2010), the effects of the dinoflagellate *Cochlodinium polykrikoides* on copepod reproduction changed from beneficial to toxic with increasing cell density. Thus, it is tempting to speculate that, due to a hormesis effect (Azzam 2011), low levels of nodularin stimulate antioxidant production in females and that it results in improved egg quality through increased allocation of antioxidants to the eggs. The positive effects of cyanobacteria on copepod egg viability and early juvenile development may also be related to unknown complementary nutrients or microelements found in *N. spumigena* but missing from *B. submarina* (Schmidt and Jónasdóttir 1997). In addition, the correlations between the oxidative status variables and reproductive parameters strongly suggest that maternal oxidative status correlates with egg viability and early offspring development, but not with egg production. This implies that, for the conditions tested, the maternal oxidative status is only reflected in the quality, but not the quantity of the offspring.

Harmful algal blooms are projected to become more frequent phenomena in the future with unknown consequences for food webs. Here, we show that toxic cyanobacteria in combination with high-quality food have a negative effect on egg production rate but a positive effect on egg viability of copepod grazers, giving no net effect on viable egg production rate. Moreover, toxic cyanobacteria also had a positive effect on the nauplii development and survival, which suggest that low levels of *N. spumigena* could be beneficial for *A. bifilosa* recruitment. These findings suggest complex effects of cyanobacteria and their toxins on reproduction and juvenile development of copepods and highlight the need for further studies. The levels of warming and acidification used in this study (Caldeira and Wickett 2003; HELCOM 2007; IPCC 2007) created significant effects on copepod oxidative status and reproduction. The results emphasize that mobile crustaceans might not be as tolerant to acidification as previously suggested (Melzner et al. 2009; Kroeker et al. 2013). However, interpretation of potential long-term responses should be made carefully, as the results are based on a short-term study and we do not know how the copepods will adapt. Future research should therefore combine different climate and other human-induced factors and use realistic future scenarios for several generations.
